# Comment on “immunosuppressive therapy management during sepsis in kidney transplant recipients: a prospective multicenter study”

**DOI:** 10.1186/s13613-025-01598-x

**Published:** 2025-10-18

**Authors:** Shuo Lin, Jianjie Ju, Zhouhua Wang

**Affiliations:** 1 The School of Clinical Medicine, Fujian Medical University, Fuzhou, China; 2 The School of Pharmacy, Fujian Medical University, Fuzhou, Fujian China; 3 Mindong Hospital Affiliated to Fujian Medical University, Ningde, Fujian China

To the editor

We read with great interest the study by Rivet et al., which prospectively examined the management of immunosuppressive therapy (IST) in kidney transplant recipients (KTR) with sepsis [1]. This timely investigation addresses a critical and often difficult clinical question, and the authors are to be commended for assembling multicenter data in such a challenging population. While we recognize the value of their findings, we would like to highlight several issues that may merit further discussion.

**First**, the infection spectrum in this cohort warrants closer consideration. A substantial proportion of patients were admitted with SARS-CoV-2 pneumonia. Given the distinct pathophysiology of viral infections and the frequent use of concomitant therapies such as corticosteroids and tocilizumab, it is uncertain whether the conclusions can be extrapolated to patients with more typical bacterial sepsis. Stratified analyses contrasting bacterial and viral sepsis would help determine whether IST management strategies should be tailored to the underlying infection type.

**Second**, the complexity of immunosuppressive regimens in clinical practice may not be fully reflected by assessing the discontinuation of single agents. Most KTR patients are treated with combinations of calcineurin inhibitors (CNI), mycophenolate (MPA), mTOR inhibitors, and corticosteroids. The clinical consequences may depend not only on withholding a single drug, but also on the interactions between drugs and the cumulative level of immunosuppression. For example, discontinuing MPA while simultaneously tapering corticosteroids may carry different implications than either adjustment alone. Future studies incorporating drug combination patterns and overall immunosuppressive burden could provide a more nuanced understanding.

**Finally**, the reported incidence of acute rejection during follow-up was low, with only two cases observed. Although reassuring, this likely underestimates the true risk of rejection, particularly in its subclinical form. Without systematic surveillance—such as protocol biopsies, donor-specific antibody (DSA) monitoring, or immune profiling—it is difficult to rule out silent immunologic injury. Such injury may contribute to chronic allograft dysfunction beyond the study’s follow-up period. We therefore encourage future work to include standardized monitoring of graft rejection to better balance infection-related risks against long-term graft preservation.

**In conclusion**, Rivet et al. provide valuable insight into the management of IST during sepsis in kidney transplant recipients, underscoring the delicate balance between infection control and graft protection. Addressing the points raised above in future studies could strengthen the applicability of these findings and inform more individualized approaches to immunosuppressive management in this vulnerable population.
